# Suprachoroidal Delivery of Small Molecules, Nanoparticles, Gene and Cell Therapies for Ocular Diseases

**DOI:** 10.3390/pharmaceutics13020288

**Published:** 2021-02-22

**Authors:** Chen-rei Wan, Leroy Muya, Viral Kansara, Thomas A. Ciulla

**Affiliations:** Clearside Biomedical, 900 North Point Parkway, Suite 200, Alpharetta, GA 30005, USA; cherry.wan@clearsidebio.com (C.W.); leroy.muya@clearsidebio.com (L.M.); viral.kansara@clearsidebio.com (V.K.)

**Keywords:** suprachoroidal, microinjector, ocular drug delivery, suprachoroidal space (SCS), gene therapy, anti-VEGF, macular degeneration, glaucoma

## Abstract

Suprachoroidal drug delivery technology has advanced rapidly and emerged as a promising administration route for a variety of therapeutic candidates, in order to target multiple ocular diseases, ranging from neovascular age-related macular degeneration to choroidal melanoma. This review summarizes the latest preclinical and clinical progress in suprachoroidal delivery of therapeutic agents, including small molecule suspensions, polymeric entrapped small molecules, gene therapy (viral and nonviral nanoparticles), viral nanoparticle conjugates (VNCs), and cell therapy. Formulation customization is critical in achieving favorable pharmacokinetics, and sustained drug release profiles have been repeatedly observed for multiple small molecule suspensions and polymeric formulations. Novel therapeutic agents such as viral and nonviral gene therapy, as well as VNCs, have demonstrated promise in animal studies. Several of these suprachoroidally-administered therapies have been assessed in clinical trials, including small molecule suspensions of triamcinolone acetonide and axitinib, viral vector RGX-314 for gene therapy, and VNC AU-011. With continued drug delivery research and optimization, coupled with customized drug formulations, suprachoroidal drug delivery may address large unmet therapeutic needs in ophthalmology, targeting affected tissues with novel therapies for efficacy benefits, compartmentalizing therapies away from unaffected tissues for safety benefits, and achieving durability to relieve the treatment burden noted with current agents.

## 1. Current Treatment Landscape and Unmet Clinical Need

Highly active research and development efforts are ongoing to optimize ocular drug delivery for potential improvement in efficacy, safety, and durability benefits. The most common ocular drug delivery method for treating posterior eye diseases is intravitreal (IVT) injections [1,2,3,4]. It is estimated that 24.4 million IVT injections were performed globally in 2019 with 6.9 million of these injections performed in the United States, most often for neovascular age-related macular degeneration (nAMD) and diabetic macular edema (DME) [5]. While IVT injections of anti-vascular endothelial growth factor (anti-VEGF) therapies are commonly performed in an office setting, and have demonstrated remarkable therapeutic benefits in controlled clinical trials [6,7,8,9,10,11,12,13,14,15,16,17], real world data on patients receiving IVT injections of anti-VEGF therapies show a much more modest improvement in patients’ vision [18,19,20,21,22]. This disparity may relate to treatment burden, with patients undertreated due to an inability to maintain the frequent treatments in fixed regimens utilized in clinical trials. Furthermore, a subset of patients responds incompletely to anti-VEGF treatments regardless of the frequency of administration [23]. Other pharmacological agents, such as corticosteroids, that are commonly injected intravitreally for posterior eye diseases, may have undesirable side effects, including ocular hypertension and/or cataracts, due to their anterior chamber exposure [24,25,26,27,28,29,30,31].

In addition to posterior eye diseases that are currently treated with IVT injections, there remain significant unmet clinical needs in other ocular disorders, such as glaucoma, that are currently treated topically with eye drops and/or surgery. Glaucoma patients face challenges including compliance with proper administration of topical eye drops of beta blockers (e.g., timolol), alpha agonists (e.g., brimonidine), Rho kinase inhibitors (e.g., netarsudil), and prostaglandin analogs (e.g., latanoprost) [32,33,34]. These drugs act by decreasing the production and/or increasing the outflow of the aqueous humor. In some instances, combination treatments may also be required to provide adequate treatment. Specifically related to topical eye drops, in addition to patient compliance, there are challenges maintaining drug concentrations above therapeutic levels due to the high clearance rate via nasolacrimal drainage and/or dilution from production of reflex tears [35,36]. Taken together, there remains an unmet clinical need for improved delivery to targeted ocular tissues, compared to current topical or intravitreal delivery methods.

One possible solution to address this unmet need is the delivery of therapeutic agents into the suprachoroidal space (SCS). The SCS is the ‘potential space’ between the sclera and choroid that circumferentially spans the entire posterior segment of the eye from the ciliary body rearwards. Potential spaces in the body are areas between directly apposed organs or tissue layers. These potential spaces, such as the pleural space, the pericardial cavity, and the SCS, expand when fluid enters and flows within the space and collapse upon fluid egress. These potential spaces can serve as “druggable” targets for the delivery of therapeutic agents.

**Figure 1:**
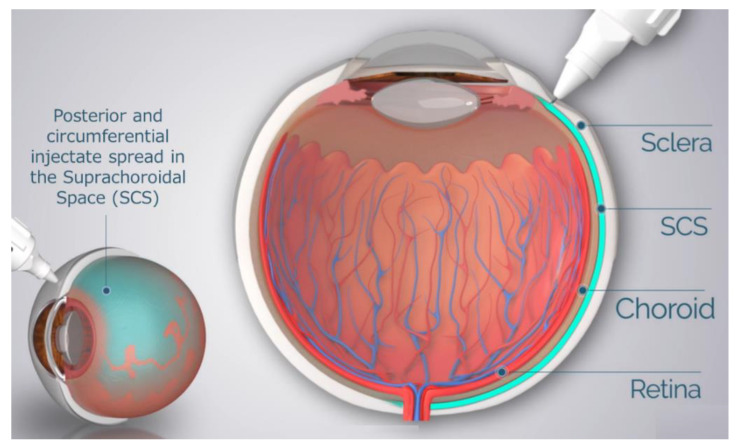
Schematic of Microneedle Injection into the Suprachoroidal Space (SCS).

Drug delivery to the SCS has unique potential advantages in that (1) it specifically targets affected chorioretinal tissues with posterior and circumferential spread of the drug administered, (2) it may provide sustained drug kinetic release profiles, depending on the physio-chemical formulation properties, and (3) it may spare the unaffected anterior segment of the eye and the vitreous chamber, thus minimizing risks associated with off-target effects to potentiate safety [37,38]. To reliably access the SCS, microneedles have been designed to be long enough to penetrate through the sclera, delivering therapeutic agents into the SCS, without penetrating into the vitreous. The therapeutic agent flows both circumferentially and posteriorly towards the back of the eye, targeting chorioretinal tissues (Figure 1). In recent years, multiple clinical trials have evaluated this technique of suprachoroidal (SC) delivery with therapeutic agents ranging from small molecule suspensions to viral vector therapies.

**Figure 2:**
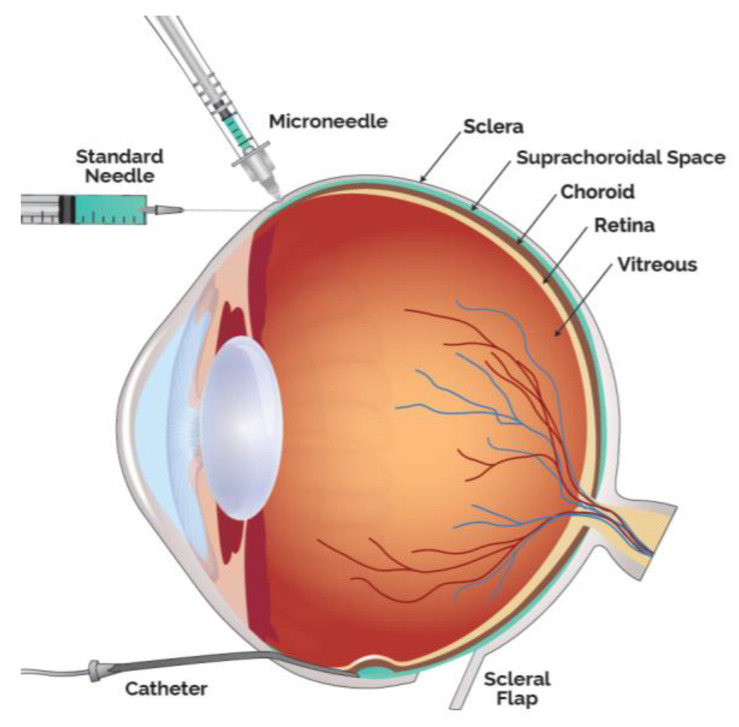
Modalities to Administer Therapeutic Agents into the Suprachoroidal Space (SCS).

Drug delivery into the SCS has been achieved in multiple ways, in addition to the microneedle method, the predominate access technique clinically utilized. Small case series have also been performed by creating a scleral flap and a suprachoroidal (SC) pocket to insert autologous tissue. Preclinically, SC injections have been achieved with scleral flap technique, catheters as well as standard hypodermic needles (Figure 2).

The objective of this review article is to summarize the current status of preclinical and clinical therapeutic agents that have been administered into the SCS. As synthesized in this review article, SC administration may offer unique advantages, such as compartmentalization away from the anterior segment and prolonged durability with appropriate formulations. Clinical trials with suprachoroidally administered triamcinolone acetonide, a small molecule suspension, have already demonstrated safety and efficacy. This route of administration may further play a significant role in ocular drug delivery of additional novel therapeutic agents for a wide range of ocular diseases, spanning from glaucoma and ocular melanoma to various common chorioretinal diseases, such as age-related macular degeneration (AMD) and diabetic retinopathy (DR).

## 2. Small Molecule Suspensions

Introduction of low-solubility therapeutic agents, such as small molecules in suspension, into the SCS is one strategy employed to promote both improved pharmacokinetic (PK) profiles and targeted drug delivery. Several well-characterized therapeutic agents, compounded as small molecule suspensions, have been administered suprachoroidally.

### 2.1. Corticosteroid

Safety and efficacy of an SC-delivered investigational formulation of triamcinolone acetonide (TA) has been evaluated in multiple controlled clinical trials [39,40,41,42,43] and its pharmacokinetic and pharmacodynamic characteristics have been evaluated in multiple animal studies.

Preclinically, favorable pharmacokinetics of SC TA has been demonstrated. This commonly used corticosteroid is a crystalline small molecule with low aqueous solubility (ranging between 0.02 mg/mL at 28 °C and 0.03 mg/mL at 50 °C [44]) and has historically been administered via topical, periocular or intravitreal routes, for the treatment of ocular inflammation. At a particle size distribution in the low micrometer range after micronization, it has been demonstrated that the concentration of TA in the SCS in various preclinical models can be maintained above therapeutic levels for an extended period of time [45].

With respect to ocular distribution, when TA was injected into the SCS in rabbits, TA concentrations in the retinal pigment epithelium (RPE)-choroid-sclera (RCS) and the retina were significantly higher than those in both the aqueous humor and the vitreous through the duration of the study (91 days) [46]. This study illustrated that drug delivery into the SCS can be compartmentalized, away from the anterior segment and the vitreous humor, preferentially targeting chorioretinal tissues and provide sustained PK profile.

Pharmacodynamically, efficacy of SC TA was supported in a porcine model of uveitis. In this model, lipopolysaccharides (LPS) were introduced into pigs to induce uveitis [47]. Suprachoroidal administration of TA meaningfully reduced inflammation over time. In an in vivo model, Chen et al. showed that TA delivered via the SC route provided excellent targeting to the posterior retina; resulting in improved efficacy with significantly fewer aqueous humor cells and lower vitreous opacity scores compared to TA administered via the sub-Tenon route in rabbits [48]. Similar studies have also shown that TA administered via the SC or IVT routes in a model of endotoxin-induced panuveitis in albino rabbits led to comparable efficacy in the reduction of ocular inflammation [49]. Furthermore, an in vivo porcine model of acute uveitis demonstrated that 1/10th the dose of TA administered suprachoroidally was as effective as the full dose administered intravitreally, with no adverse effects [47]. Using a similar porcine model, another study showed that TA administered suprachoroidally was more effective in reducing ocular inflammation than low dose oral prednisone (0.1 mg/kg/day) administered for three days, and resulted in an improved rate of reduction in inflammation when compared to high dose oral prednisone (1 mg/kg/day) [50]. These results validate SC administration as a potential drug delivery route for small molecule suspensions.

**Figure 3:**
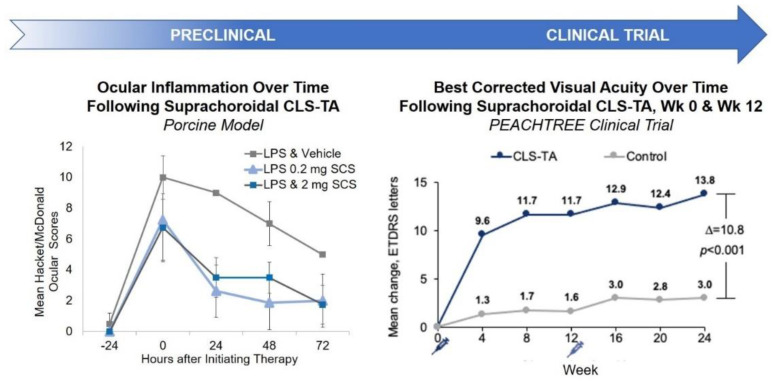
Preclinical and Clinical Results of SC Administration of Small Molecule Suspensions. CLS-TA: investigational formulation of triamcinolone acetonide developed by Clearside Biomedical (Alpharetta, GA, USA); LPS: lipopolysaccharides.

These preclinical study results translated well into clinical trial outcomes. Specifically, in a phase 3 double-masked and randomized clinical trial for macula edema associated with noninfectious uveitis, 46.9% of the subjects treated with two SC injections of TA administered 12 weeks apart gained at least 15 Early Treatment Diabetic Retinopathy Study (ETDRS) letters from baseline in best corrected visual acuity (BCVA) at 24 weeks, compared to 15.6% in the sham control group [39] (Figure 3).

### 2.2. Tyrosine Kinase Inhibitor

Another small molecule suspension that has been evaluated for potential SC delivery is axitinib. Axitinib has several properties with attractive therapeutic potential. It is a highly potent tyrosine kinase inhibitor (TKI) with pan-VEGF inhibition and high binding affinities [51]. Pan-VEGF inhibition may have benefits over current specific VEGF-A inhibition for the treatment of nAMD, DR, and DME, as other VEGF ligands such as VEGF-C and VEGF-D, have been shown to be upregulated after anti-VEGF-A administration both locally, after the treatment of nAMD, and systemically [52,53]. This upregulation of other VEGF ligands could contribute to tachyphylaxis, a form of treatment resistance, and lead to refractory cases clinically. Axitinib has also been shown to more effectively inhibit angiogenic sprouts than anti-VEGF-A inhibition in an in vitro angiogenesis model [54]. One recent phase 2 clinical trial demonstrated that broad VEGF inhibition yielded a statistically significantly better visual outcome in nAMD than focused VEGF-A inhibition [55]. In addition, axitinib is a more highly potent TKI than others that have undergone assessment in ocular clinical trials [56] and has demonstrated more potent inhibition of murine corneal neovascularization, compared to other TKIs at the same dose [57]. Axitinib has been shown to not only inhibit angiogenesis, but also regress established neovascularization in a preclinical choroidal neovascularization model, which may be more relevant to potential clinical use [58]. Finally, axitinib has shown better biocompatibility with ocular cells, including retinal pigment epithelial cells, compared to other TKIs in an in vitro study, suggesting the potential for intrinsic safety benefits [59].

While axitinib, as a poorly water-soluble small molecule (molecular weight: ~387 g/mol, solubility: 0.2 µg/mL in neutral pH aqueous media [60]), may not be well-suited for many forms of ocular delivery, it can be injected into the SCS as a stable suspension. In a rabbit PK and ocular tolerability study, a single bilateral SC injection of an axitinib suspension at 2 different doses (either 0.03 or 0.1 mg per eye) was administered to Dutch-Belted rabbits [61]. Axitinib was well-tolerated and optical coherence tomography (OCT) showed no evidence of choroidal/retinal degeneration through the duration of the study (10 weeks). More importantly, efficacious and sustained levels of axitinib, above the in vitro IC_50_ for the VEGF 2 receptor, were observed in the posterior ocular tissues—specifically in the RCS and retina—throughout the duration of the study, while no axitinib was detected in the plasma or aqueous humor. Furthermore, when compared to an equivalent dose of IVT axitinib in this rabbit model, SC axitinib resulted in RCS levels that were 11 times greater, supporting the potential for favorable targeting of chorioretinal tissues, which are affected in common causes of vision loss, such as AMD and DME. In a separate rabbit PK and ocular tolerability study, a single SC axitinib suspension was administered at a dose of 4 mg/eye. Over the duration of this 91-day study, axitinib was quantifiable at all timepoints in the RCS, retina and the vitreous humor with the highest concentration detected in the RCS. The administered dose was generally well tolerated. Currently, a phase 1/2a clinical trial of suprachoroidally-administered axitinib, for the treatment of nAMD, is underway [62].

### 2.3. Complement Inhibitor & Plasma Kallikrein Inhibitor

Additional small molecules have been investigated to target other signaling pathways, such as the complement system, a potential treatment target for non-neovascular age-related macular degeneration (dry AMD). Suprachoroidally administered A01017 (Achillion Pharamaceuticals, Blue Bell, PA, USA now AstraZeneca, Cambridge, UK), a potent small molecule complement factor D inhibitor [63], was well tolerated with sustained drug levels in posterior segment tissue of rabbits for up to 92 days. Similar to TA and axitinib, a high level of A01017 was observed in the RCS and retina throughout the entire duration of the study with an estimated half-life of approximately 66–76 days. Low or no quantifiable A01017 was detected in the vitreous humor, aqueous humor or plasma.

Yet another example of a small molecule suspension that has demonstrated preclinical ocular tolerability and sustained ocular drug levels is BCX4161 (BioCryst Pharmaceuticals, Durham, NC, USA), a potent and selective inhibitor of human plasma kallikrein, that is elevated in patients with diabetic macular edema [64]. BCX4161 was suprachoroidally administered to Dutch-Belted rabbits and was found to be well tolerated with sustained levels detected throughout the RCS and both central and peripheral retina over the 12-week study duration. Furthermore, the concentration of BCX4161 in the retina was 1 to 2 orders of magnitude higher than levels in the vitreous humor.

Collectively, these study results demonstrate that insoluble small molecule suspensions delivered via the SC route result in targeted high levels in chorioretinal tissues with potential efficacy benefits, compartmentalization away from unaffected tissues for potential safety benefits, and durability to potentially relieve treatment burden compared to current IVT therapies (Table 1).

## 3. Polymeric Formulations

### 3.1. Corticosteroid

An additional approach to enhancing the durability of a therapeutic agent in the SCS is through polymeric entrapment of the active pharmaceutical ingredient (API). A sustained release formulation of dexamethasone, combined with a Laponite carrier, has been evaluated in rabbits [65,66]. Laponite is a synthetic clay used as a drug delivery carrier and has been demonstrated to be safe and biocompatible in vivo [67]. In this rabbit study, the dexamethasone/Laponite (DEX/LAP) formulation was administered suprachoroidally and dexamethasone levels were evaluated for up to 24 weeks. In the choroid and retina, dexamethasone concentration peaked at 1 week and a detectable concentration was maintained throughout the duration of the study, with an estimated half-life of 36.4 days.

Dexamethasone has also been incorporated into polyurethane implants and evaluated in a rat model of endotoxin-induced uveitis [68]. The sheet-like implants were implanted into the SCS, 4 mm posterior from the limbus and were found to be safe and biocompatible. The drug release profile, assessed in an in vitro release model, revealed a burst of dexamethasone the first week followed by a gradual sustained release as a function of the polymeric degradation, for up to 42 days. This release profile elicited an in vivo response, with a superior suppression of the inflammatory response, indicated by a reduction of the infiltration of polymorphonuclear cells and macrophages in the dexamethasone-laden implants, compared to placebo implants.

### 3.2. Neuroprotection

Furthermore, tauroursodeoxycholic acid (TUDCA), a potential neuroprotective agent, has been evaluated when administered suprachoroidally [69,70]. Olsen et al. loaded polycaprolactone-polyethylene glycol polymer with TUDCA and implanted it into the SCS or the vitreous humor (IVT) in pigs [71]. In in vitro drug release assays, a rapid burst of TUDCA from the implant surface was observed, followed by a slower, sustained release through diffusion. In vivo analysis revealed that higher serum levels of TUDCA were detected at week 2 and week 4 with SCS TUDCA implants, compared to IVT implants. Furthermore, TUDCA concentration rapidly decreased from week 1 to week 4 in the peri-retina and peri-choroid tissues and the levels remained low in the neurosensory retina through the duration of the study for the SCS TUDCA implants. These results suggest that while the TUDCA concentration was measurable in local ocular tissues for the SCS group, TUDCA may be rapidly cleared through the choroidal vasculature. Additional optimization may be required to slow the release of TUDCA from the implant.

### 3.3. Hypoxia-Inducible Factor (HIF) Inhibitor

Another polymer that has been used as a controlled drug release platform is poly (lactic-co-glycolic acid) (PLGA). Hackett et al. incorporated acriflavine (ACF), a highly aqueous-soluble molecule (solubility: 0.33 g/mL [72]), into PLGA and introduced the ACF-PLGA microspheres into the vitreous or the SCS in rats and rabbits [73]. The function of ACF is to inhibit HIF-1, a potent angiogenic factor that has been implicated in the pathogenesis of choroidal neovascularization [74,75]. By incorporating ACF into PLGA microparticles, the drug release profile was extended to up to 60 days in vitro. SC injections of the ACF-PLGA microparticles in rabbits were safe as demonstrated by fundus imaging, electroretinogram (ERG), intraocular pressure (IOP) monitoring, and retinal histology. SC injections of the ACF-PLGA microparticles suppressed laser-induced choroidal neovascularization for at least 18 weeks in Male Norway Brown rats.

### 3.4. Alpha-2-Agonist

Poly(lactic acid) microspheres loaded with brimonidine have also been introduced into the suprachoroidal/superciliary space to evaluate its impact on IOP [76]. Brimonidine, an alpha-2 adrenergic agonist, is a commonly used anti-ocular hypertensive treatment and is typically administered as a thrice daily eye drop. Chiang et al. developed microspheres with a sustained release profile of brimonidine and evaluated the effect of this formulation in the SCS in New Zealand White rabbits. The microspheres were suspended in a viscous fluid (5% carboxymethyl cellulose in Hank’s buffer salt solution) to localize the drug close to the injection site, 3 mm posterior to the limbus. The authors demonstrated IOP reductions of up to 6 mm Hg for up to 1 month, thus providing evidence of feasibility of sustained IOP reduction with brimonidine-laden microspheres delivered suprachoroidally.

### 3.5. In Situ Forming Hydrogel

Lastly, the administration of an in situ forming hyaluronic acid (HA) hydrogel into the supraciliary/suprachoroidal space has been shown to reduce IOP in normotensive rabbits for more than 4 months [77]. The IOP reduction was found to strongly correlate with the SCS expansion, facilitating the uveoscleral outflow pathway. This is a potential novel drug-free, surgery-free option for sustained IOP reduction.

Taken together, utilization of polymers to incorporate therapeutic agents is a promising method for sustained release of drug in the SCS (Table 2). It is foreseeable that the release profile may be tunable based on polymeric formulation customization. 

## 4. Viral and Nonviral Gene Therapies

The standard for ocular gene therapy administration is subretinal injection, a highly-specialized surgical procedure, which is generally performed at selected Centers of Excellence, given the potential complications of pars plana vitrectomy associated procedures such as retinotomy [78,79]. Suprachoroidal gene delivery, an alternative route of administration, has demonstrated promise preclinically and is currently undergoing clinical evaluations [80]. Delivery of gene therapy via SC injection enables an in-office gene therapy administration and may facilitate increased patient access as well as reduced procedural risks compared to subretinal delivery. The potential of SC gene therapies using marker genes, such as green fluorescent proteins (GFP) and luciferase, has been studied preclinically [80,81,82]. Briefly, multiple animal studies have demonstrated proof of concept with GFP expression after SC injections of adeno-associated viral vector (AAV) and nonviral vectors expressing GFP [83,84,85,86,87,88,89,90]. In addition, the application of nanoparticle systems to deliver drugs or gene therapy into the SCS may further enhance therapeutic potential by optimizing for durability or particle spread [91,92]. This review article will focus mainly on the latest studies using transgenes expressing active therapeutic agents.

### 4.1. Viral Vector Gene Therapy

Ding et al. evaluated suprachoroidally administered AAV expressing an anti-VEGF protein (RGX-314, REGENXBIO, Rockville, MD) in a VEGF-induced vascular leakage model in rats [86]. In this study, Brown Norway rats were dosed with RGX-314, either suprachoroidally or subretinally, in one eye and placebo vehicle formulation in the fellow eye. In this model, eyes that had previously been treated with RGX-314 either suprachoroidally or subretinally exhibited normal vasculature on fundus imaging, while the fellow RGX-314 untreated eye showed vessel leakage, dilation, and retinal hemorrhage. There were similar levels of suppression of VEGF-induced vascular leakage in the rats dosed with RGX-314 suprachoroidally or subretinally. A Phase 2 clinical trial for nAMD with SC administration of RGX-314 is currently underway, with initial safety data expected in early 2021 [93,94]. A similar phase 2 trial of SC injected RGX-314 for DR has been initiated, with preliminary data expected in 2021 [94].

Han et al. recently assessed retinal tropism and transduction efficiency of five different AAV serotypes expressing GFP driven by a cytomegalovirus (CMV) promoter, after intravitreal, subretinal, or suprachoroidal delivery in rats [85]. Suprachoroidal delivery yielded similar outcomes as subretinal delivery for all AAV serotypes, but resulted in a wider distribution and greater outer nuclear layer transduction. The authors postulate that vectors may cross Bruch’s membrane to transduce the retina and that suprachoroidal delivery may be a viable route for AAV-mediated retinal transduction. The authors also suggested further studies to evaluate serotype-specific differences in tropism in large animals and to evaluate the transduction efficiency in animal models of retinal degeneration.

### 4.2. Nonviral Vector Gene Therapy

Nanoparticles (NPs) have also been evaluated as a non-viral-based gene therapy. Kansara et al. administered rod-shaped and ellipsoid-shaped luciferase DNA NPs (DNPs) as a unilateral dose in New Zealand White rabbits in a study to assess ocular tolerability and transfectability [81]. Results from this study support that luciferase DNPs administered via the SC and subretinal routes resulted in comparable transfection of chorioretinal tissues. Both rod-shaped and ellipsoid-shaped DNPs were well tolerated in rabbits [95].

Shen et al. administered NPs containing a reporter gene into the SCS and noted expression in rat photoreceptors and RPE throughout the entire eye for at least 8 months, with multiple injections further increasing expression. When they administered a VEGF-expressing plasmid into the SCS in rats, severe neovascularization and subsequent subretinal fibrosis were observed at 1 and 5 months, respectively [87]. Conversely, when plasmid encoding VEGF-neutralizing protein was administered into the SCS, VEGF-induced vascular leakage and neovascularization were significantly reduced. In this study, the NPs were on the order of 200 nm, larger than AAVs, which may promote longer retention of the NPs in the SCS [96,97]. These studies further suggested that nonviral NP-based gene therapy has potential advantages over viral vector-based gene therapy, in that NPs are less immunogenic, which may facilitate repeat dosing and customizable therapy as needed. Similarly, higher therapeutic vector-genome doses may also be delivered using NPs to improve transfection efficiency and DNPs can deliver larger-sized clinically relevant genes compared to viral vector-based gene therapy [80].

### 4.3. Viral Nanoparticle Conjugate

Additional particles such as viral nanoparticle conjugates (VNCs) in the SCS have been investigated for choroidal melanoma [98]. One such VNC is AU-011 (Aura Biosciences, Cambridge, MA, USA), a therapeutic light-activated VNC, modeled on Human Papilloma Virus, that binds selectively to cancer cells through modified heparan sulphate proteoglycans. When AU-011 is photoactivated with non-thermal infrared laser light, it induces cell membrane disruption and cancer cell necrosis. Anti-tumor effects of AU-011 in the SCS were evaluated in a rabbit model. In this model, human melanoma cells were implanted into the SCS, developing into xenogeneic tumors in rabbits. AU-011 was then injected suprachoroidally and activated with infrared laser light. In this study, tumor regression was observed in all rabbits [98]. This study expands the horizon of therapeutic agents that can be introduced into the SCS with potentially promising safety and efficacious pharmacological profiles. Suprachoroidally administered AU-011 is currently undergoing phase 2 clinical investigation for patients with choroidal melanoma [99].

The existing preclinical studies revealed that SC delivery of viral and nonviral gene therapies is a viable treatment route of administration and the ongoing clinical studies will further demonstrate its potential (Table 3). 

### 4.4. Nanoparticles

A plethora of nanoparticles of different nature has been administered into the SCS. In addition to the viral and nonviral nanoparticles described in the previous section, additional inorganic and polymeric particles have also been evaluated within the SCS (Table 4).

## 5. Proteins and Peptides

### 5.1. Anti-VEGF

Another class of biologics that has been suprachoroidally administered includes peptides and proteins. Anti-VEGF therapy is commonly administered intravitreally, but no anti-VEGF therapy has been approved for administration into the SCS. Olsen et al. compared intravitreal and SC injections of bevacizumab in a porcine model [104]. From a safety perspective, no findings were observed for the SC group, while vitritis and granulomatous vasculitis were noted in 7 of 30 eyes in the intravitreal group. Given bevacizumab is frequently administered intravitreally in humans without ocular inflammation, the authors hypothesized that the introduction of a human antibody into porcine vitreous humor may result in a xenograft immune rejection. Pharmacokinetically, bevacizumab concentrations decreased gradually over 60 days in the intravitreal group while bevacizumab levels dropped off rapidly and were no longer detected after 7 days in the SC group. Disparate protein distribution patterns, corresponding to the expected levels depending on the routes of administration, were also observed for the two groups; while bevacizumab was primarily located within the inner retina and the internal limiting membrane in the intravitreal group, bevacizumab was mostly found in the RCS (dose depot) for the SC group. Taken together, these data suggest that rapid clearance of suprachoroidally injected bevacizumab, a soluble protein, may be occurring via the choroidal vasculature or other fluid outflow mechanisms such as the uveoscleral outflow pathway. However, the higher levels observed in the choroid after SC administration could yield some efficacy benefits in treating choroidal neovascularization, and further study with sustained release polymeric formulations is warranted.

Bevacizumab was also evaluated in the SCS in 8-week-old Lewis rats [105]. Saline (control group) or 0.2 mg of bevacizumab (treatment group) were injected into the SCS. Retinal thickness and choroidal vasculature were observed with histology after one month. Compared to the control group, reduced vascular area was observed in the choroidal vasculature in the treatment group. No difference in retinal morphology (retinal thickness, outer nuclear layer thickness) was observed. This study indicates that SC bevacizumab was able to elicit a biological response (i.e., reduced choroidal vascular area) with acceptable safety profile (i.e., normal retinal morphology).

Efficacy of another anti-VEGF agent, aflibercept, has also been investigated suprachoroidally in a laser-induced choroidal neovascularization model in rats [106]. Either aflibercept or saline was injected into the SCS and the area of neovascularization was measured after 3 weeks. Aflibercept-treated animals had significantly reduced neovascularization, compared to the saline control group. This result echoes the previous bevacizumab findings.

### 5.2. Vasoconstrictor

Another protein that has been administered into the SCS is endothelin-1, a potent vasoconstrictor that has been previously investigated as a model for optic nerve ischemia [107,108,109]. Nork et al. injected endothelin-1 into the SCS in rabbits as a model to reduce choroidal blood flow [110]. Compared to the vehicle control, endothelin-1 injected eyes had significantly lower choroidal blood flow. This study demonstrated that endothelin-1 in the SCS was able to elicit a targeted physiological response, measured by choroidal blood flow assessment. This study, in conjunction with an early complementary model of inner retinal ischemia after IVT endothelin-1 administration, illustrates the ability to target pharmacologic effect to ocular compartments, based on the route of administration [111].

As highlighted previously, one of the limitations of utilizing soluble proteins and peptides is that they are dispersed in an aqueous state, which facilitates rapid clearance therefore limiting their duration in the SCS, compared to insoluble small molecule therapeutic suspensions. Further formulation development is warranted to optimize durability of these biologics within the SCS, and one such effort is already underway with a hydrogel formulation of aflibercept currently undergoing preclinical assessment [112]. Another potential solution to enhance durability and improve retinal exposure is to leverage the advancements in nanoparticle systems, such as liposomes, lipid nanoparticles or lipid nanoparticles containing cell-penetrating peptide sequences, to encapsulate therapeutic agents and to target chorioretinal tissues [113,114,115].

## 6. Cell Therapy

### 6.1. Autologous Tissue–Trophic Factors

Beyond gene and protein-based therapies, cells and autologous tissue derivates have also been evaluated in the SCS for retinal diseases. These potential therapies include platelet rich plasma (PRP) and stromal vascular fractions (SVFs). Limoli et al. conducted multiple small clinical evaluations, placing autologous fat tissue, SVF, and PRP in the SCS through a deep 5 × 5 mm sclerotomy window 8 mm post-limbus [116]. The rationale for the tissue implantation was that SVF and PRP both contain growth factors that may play a role in promoting retinal health through paracrine signaling [117,118] and adipose-tissue-derived stem cells, albeit a small fraction of the cell population, have been found in SVFs [119]. In the first study, 12 eyes of 12 patients with dry AMD underwent the cell transplantation procedure; ERGs and BCVA were used as efficacy indicators. Scotopic rod-ERG response was found to be statistically different between baseline and 30 days post treatment. No statistical differences could be identified for BCVA, maximal rod-cone-ERG, or photopic cone-ERG [116]. The treatment was well-tolerated.

To further investigate the effect of the autologous tissue implantation, the authors conducted another study with 36 eyes in 25 patients with dry AMD [120,121]. Improvements in BCVA from baseline to 6 months post procedure were observed for patients with baseline retinal thickness greater than 250 µm. The authors hypothesized that the thicker retina may be indicative of higher cellularity in response to the paracrine signaling effects from the autologous tissue. No adverse events were documented. This treatment has also been evaluated for 34 eyes in 25 patients with retinitis pigmentosa (RP) [122,123]. No statistically significant improvements from baseline in BCVA to 6 months were observed.

### 6.2. Allogenic Cell Therapy

The same technique of transplanting autologous tissue has also been utilized for implanting mesenchymal stem cells derived from umbilical cords (UC-MSCs) [124] or adipose tissues (AD-MSCs) [125,126]. Twenty-nine eyes in 23 patients with optic atrophy were enrolled at a single center, received five million UC-MSCs per eye, and followed for a minimum of 12 months. The treatment was well-tolerated and improvements in visual field and BCVA were observed between baseline and 12 months.

While cell-based treatment yielded no serious safety signals in these initial small studies, the milieu of cytokines in PRP, SVF and cell-based products requires additional study to better understand the mechanism of action and characterize the safety, and when appropriate, to evaluate efficacy with a randomized clinical trial [127]. Given the unfortunate history of permanent vision loss after “stem cell” clinic treatment of AMD patients with unapproved cell-based products, the safety and efficacy of cell-based products for ocular diseases must be assessed through institutional review board (IRB) approved clinical trials [128].

Collective summary of the preclinical and clinical investigations of proteins, peptides and cell therapies is described in Table 5.

## 7. Conclusion and Future Perspective

An increasing number of preclinical and clinical investigations involve the delivery of existing and novel therapeutic agents into the SCS, the potential space between the sclera and choroid and a promising drug delivery route to the posterior segment of the eye.

The durability of therapeutic agents in the SCS appears to be highly dependent on the formulation attributes; while poorly aqueous soluble small molecule suspensions maintain a sustained drug concentration gradient in the target ocular tissues, highly soluble small molecules and proteins are rapidly cleared from the SCS through the choroidal vasculature. To improve durability of these highly soluble therapeutic agents, further studies with sustained release polymeric formulations or nanoparticles systems are underway. Proof of concept evaluations of novel biologics-based therapeutics have yielded promising preclinical outcomes, including transduction and transfection of chorioretinal tissues with viral and nonviral gene therapies, as well as reduction of the size of choroidal melanomas with VNCs. Suprachoroidal drug delivery is a promising drug delivery platform, with additional ongoing preclinical and clinical studies.

### Expert Opinion

- Suprachoroidal drug delivery is a promising route of administration for ocular diseases. Taking advantage of the natural ocular anatomy, suprachoroidal delivery targets high level of therapy to affected chorioretinal layers for potential efficacy benefits while compartmentalizing them away from unaffected anterior structures for potential safety benefits.
- Numerous therapies have been assessed preclinically for suprachoroidal delivery, including small molecule suspensions, polymeric formulations, and biologics including gene therapy, proteins and peptides, and cell therapy. Small molecule suspensions and polymeric formulations injected suprachoroidally have the potential for favorable pharmacokinetics with prolonged durability.
- As clinical proof of concept, the safety and efficacy of suprachoroidal delivery of an investigational suspension formulation of triamcinolone acetonide has been demonstrated in a Phase 3 clinical trial. Clinical evaluation of additional therapeutic agents, from tyrosine kinase inhibitor to gene therapy and viral nanoparticle conjugates, are underway.
- Durability of suprachoroidal drug delivery is dependent on the formulation. While soluble proteins have been shown to be rapidly transported out of the suprachoroidal space through the choroidal vasculature and uveoscleral outflow pathways, small molecules in suspension form have repeatedly demonstrated efficacious drug concentrations (above IC_50_) in target ocular tissues.
- A synergistic effort to design drug formulation tailored for suprachoroidal delivery may optimize drug delivery efficacy without compromising safety. Drug formulation designs for hydrophilic therapeutic agents that leveraging polymeric or lipid-based drug delivery system may further enhance the pharmacokinetic profiles.

## Figures and Tables

**Table 1:** Summary of Preclinical and Clinical Investigations of Small Molecule Suspensions Administered into the Suprachoroidal Space.

Therapeutic Agent	Therapeutic Class	Disease	Phase (Species)	Duration	Key Results	Ref
Triamcinolone Acetonide (TA) Suspensions	Corticosteroid	Macula Edema Associated with Uveitis	Phase 3 Clinical Study	24 weeks	In a phase 3 clinical trial for macula edema associated with noninfectious uveitis, 46.9% of the subjects treated with 2 SC injections of TA gain at least 15 ETDRS letters from baseline at 24 weeks, compared to 15.6% in the sham group	[39]
Axitinib Suspensions	Tyrosine kinase inhibitor	nAMD	Preclinical (Rabbit)	10 weeks	Efficacious and sustained level of axitinib, above IC_50_, was observed in the posterior ocular tissues throughout the duration of the study	[61]
A01017	Complement inhibitor	non-neovascular AMD	Preclinical (Rabbit)	92 days	High level of A01017 was observed in the RCS and retinal tissues throughout the entire duration of the study.	[63]
BCX4161	Plasma kallikrein inhibitor	DME	Preclinical (Rabbit)	12 weeks	BCX4161 was well tolerated and sustained levels were observed through the RCS, peripheral and central retinal tissues throughout the duration of the study	[64]

Abbreviations: SC: suprachoroidal; ETDRS: early treatment diabetic retinopathy study; TA: triamcinolone acetonide; nAMD: neovascular age-related macular degeneration; IC_50_: half maximal inhibitory concentration; RCS: RPE-choroid-sclera; DME: diabetic macular edema.

**Table 2:** Summary of Preclinical and Clinical Investigations of Small Molecules in Polymeric Formulation Administered into the Suprachoroidal Space.

Therapeutic Agent	Therapeutic Class	Disease	Phase (Species)	Duration	Key Results	Ref
Dexamethasone in Laponite	Corticosteroid	Uveitis	Preclinical (Rabbit)	24 weeks	Dexamethasone concentration peaked at 1 week and detectable concentration was maintained in the choroid and retina throughout the duration of the study	[65]
Dexamethasone in polyurethane	Corticosteroid	Uveitis	Preclinical (Rat)	42 days	Suppression of inflammatory response was observed for the dexamethasone-laden implants, compared to implants without drug	[68]
TUDCA in PCL-PEG	Neuroprotection	Retinal Degeneration	Preclinical (Porcine)	4 weeks	TUDCA concentration rapidly decreased from week 1 to week 4 in the peri-retina and peri-choroid tissue and the levels remained low in the neurosensory retina through the duration of the study	[71]
Acriflavine (ACF) in PLGA	HIF inhibitor	nAMD	Preclinical (Rat)	18 weeks	SC injections of the ACF-PLGA microparticles suppressed choroidal neovascularization for at least 18 weeks	[73]
Brimonidine in PLA	Alpha-2-agonist	Glaucoma	Preclinical (Rabbit)	1 month	IOP reductions up to 6 mmHg for up to 1 month	[76]
Hyaluronic Acid	In situ forming hydrogel	Glaucoma	Preclinical (Rabbit)	4 months	IOP reductions for up to 4 months. IOP reduction was strongly correlated with SCS expansion	[77]

Abbreviations: TUDCA: tauroursodeoxycholic acid; PCL-PEG: polycaprolactone-polyethylene; HIF: hypoxia-inducible factor; PLGA: poly lactic-co-glycolic acid; IOP: intraocular pressure; PLA: polylactic acid.

**Table 3:** Summary of Preclinical and Clinical Investigations of Gene Therapies Administered into the Suprachoroidal Space.

Therapeutic Agent	Therapeutic Class	Disease	Phase (Species)	Duration	Key Results	Ref
RGX-314	Viral vector gene therapy	nAMD DR	Preclinical (Rat)	2 weeks	Suprachoroidally administered RGX-314 was able to successfully transduce the protein fragment and have a biological effect in reducing vascular leakage. *Note: phase 2 clinical trials are currently ongoing*	[86]
VEGF-expressing plasmid NP	Nonviral vector gene therapy	Disease model for nAMD	Preclinical (Rat)	5 months	Severe neovascularization and subsequent subretinal fibrosis were observed at 1 and 5 months, respectively	[87]
VEGF neutralizing plasmid NP	Nonviral vector gene therapy	nAMD	Preclinical (Rat)	2 weeks	VEGF-induced vascular leakage and neovascularization were significantly reduced	[87]
AU-011	Viral nanoparticle conjugate	Choroidal Melanoma	Preclinical (Rabbit)	3 weeks	Tumor regression was observed in all AU-011 treated rabbits *Note: phase 2 clinical trial is currently ongoing*	[98]

Abbreviations: nAMD: neovascular age-related macular degeneration; DR: diabetic retinopathy; NP: nanoparticles; VEGF: vascular endothelial growth factor.

**Table 4:** Summary of Nanoparticle Systems Delivered into the Suprachoroidal Space.

Reporter	Nanoparticle System	Particle Size	Phase (Species)	Duration	Key Results	Ref
GFP	AAV	~25 nm	Preclinical (Mouse)	6 weeks	Transduction of the ciliary body, retinal ganglion cells, outer retina and RPE was observed	[88]
GFP	AAV	~25 nm	Preclinical (Rabbit)	6 weeks	Transduction in RPE, photoreceptors and retinal ganglion cells was observed.	[89,90]
GFP	AAV	~25 nm	Preclinical (Rat, NHP, Pig)	21 days	GFP expression observed in peripheral RPE and photoreceptors	[86]
GFP	AAV	~25 nm	Preclinical (NHP)	3 months	GFP expression observed in peripheral RPE	[84]
GFP	AAV	~25 nm	Preclinical (Rat)	2 weeks	GFP expression observed in RPE and outer nuclear layer	[85]
Luciferase	DNA-NP	10–185 nm	Preclinical (Rabbit)	1 week	High luciferase activity was measured in the retina, and RCS at day 7	[95]
Near infra-red fluorescent IO NPs	Inorganic	20 nm	Preclinical (Rat)	30 weeks	Particles were well tolerated and can be tracked by MRI for up to 30 weeks without adverse effects on retinal structures	[100]
Fluorescent Microspheres	Polymeric	1–200 nm	Preclinical (Rabbit, Ex Vivo, In Vivo)	Up to 3 weeks	Particle size did not affect particle spread distribution in the SCS. Anatomical barrier, such as the LPCA, affected spread	[96,101]
Fluorescent Microspheres	Polymeric with iontophoresis	20 nm	Preclinical (Rabbit, Ex Vivo, In Vivo)	1 week	Negatively charged nanoparticle distribution within the SCS affected by iontophoresis direction	[102]
Fluorescent Microspheres	Polymeric	20 nm–10 µm	Preclinical (Rabbit, Ex Vivo, In Vivo)	112 days	Particle size has no significant effect on SCS coverage area	[103]

Abbreviations: GFP: green fluorescent protein; AAV: adeno-associated virus; RPE: retinal pigment epithelium; NP: nanoparticle; RCS: RPE-choroid-sclera; IO: iron oxide; MRI: magnetic resonance imaging; SCS: suprachoroidal space; LPCA: long posterior ciliary artery.

**Table 5:** Summary of Preclinical and Clinical Investigations of Proteins, Peptides, and Cells Administered into the Suprachoroidal Space.

Therapeutic Agent	Therapeutic Class	Disease	Phase (Species)	Duration	Key Results	Ref
**Protein/Peptides**						
Bevacizumab	Anti-VEGF	nAMD	Preclinical (Porcine)	60 days	Bevacizumab levels dropped off rapidly and were no longer detected after 7 days in the SC group	[104]
			Preclinical (Rat)	1 month	Reduced neovascular area was observed in the choroidal vasculature	[105]
Aflibercept	Anti-VEGF	nAMD	Preclinical (Rat)	3 weeks	Aflibercept-treated animals had significantly reduced area of neovascularization in a laser-induced choroidal neovascularization model	[106]
Endothelin-1	Vasoconstrictor	Model for Retina Ischemia	Preclinical (Rabbit)	10–20min	Compared to vehicle control only, endothelin-1 injected eyes had significantly lower choroidal blood flow	[110]
**Cells/Autologous Tissue**						
PRP/SVF	Autologous tissue–trophic factors	Non-neovascular AMD	Clinical (case series)	Up to 6 months	For patients with non-neovascular AMD and retinal thickness greater than 250 µm, an improvement in BCVA was observed.	[120]
		RP	Clinical (case series)	Up to 6 months	No statistically significant improvement in BCVA from baseline to 6 months was observed.	[123]
UC-MSC	Allogenic cell therapy	Optic atrophy	Clinical (case series)	12 months	Statistically significant improvements were observed for BCVA and visual field results between baseline and 12 months after UC-MSC implantation.	[124]

Abbreviations: nAMD: neovascular age-related macular degeneration; RP: retinitis pigmentosa; BCVA: best corrected visual acuity; PRP: platelet rich plasma; SVF: stromal vascular fraction; UC-MSC: umbilical cord-derived mesenchymal stem cells.
